# Zoonotic parasitic diseases in South America: why early-career researchers matter

**DOI:** 10.1017/S0031182025100450

**Published:** 2025-07

**Authors:** Carolina De Marco Verissimo

**Affiliations:** Department of Zoology, University of Galway, Institute for Health, Discovery and Innovation (IHDI), College of Science and Engineering, Galway, Ireland

**Keywords:** early-career researchers, Global South, international collaboration, Latin America, One Health, parasites, zoonosis

## Abstract

**Figure:**
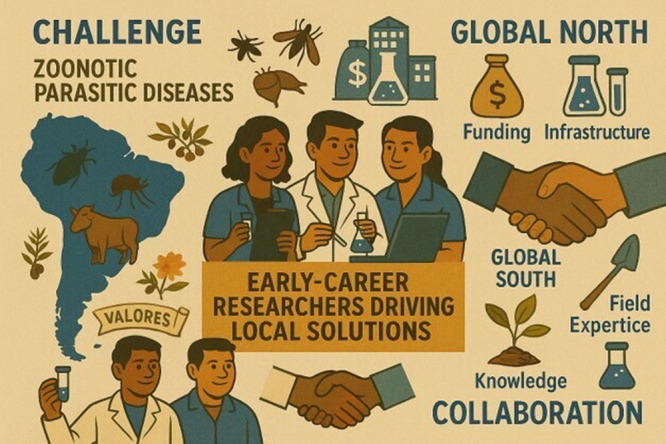

## The enduring burden of zoonotic parasitic diseases in South America

Zoonotic parasitic diseases – those transmitted between animals and humans – pose a persistent and disproportionate health and economic burden across the Global South. More than 4 billion people worldwide are at risk of at least 1 parasitic infection, with the heaviest concentrations in regions marked by poverty, inadequate sanitation, unsafe water, weak healthcare systems and intense human–animal interaction (World Health Organization, 2012, 2023a). In South America, the burden of zoonotic parasitic diseases is both historical and evolving. *Trypanosoma cruzi*, the protozoan parasite that causes Chagas disease, remains endemic despite decades of control efforts (Cucunubá et al., 2024). *Plasmodium vivax*, once considered to cause a mild malaria, continues to spread and cause significant morbidity and mortality across the Amazon basin (Russell et al., 2006). Similarly, *Taenia solium*–induced neurocysticercosis remains a leading cause of epilepsy in Andean communities of Ecuador (Cruz et al., 1999; Global Burden of Disease Collaborative Network, GBD, 2019; Centre for Disease Control and Prevention, CDC, 2022). New and emerging threats, such as zoonotic *Plasmodium simium* infections, are further challenging our understanding of parasite transmission and host dynamics (da Silva et al., 2024).

**Figure 1.**
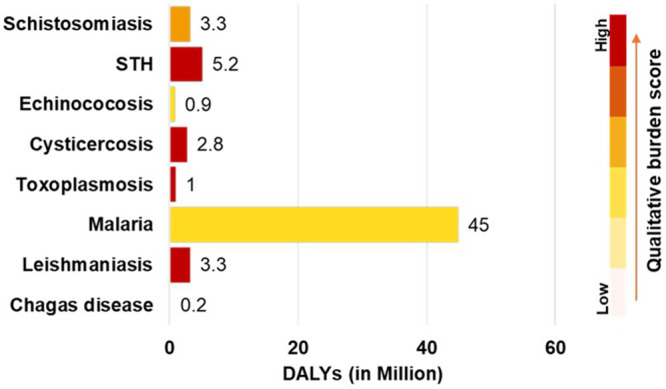
Qualitative burden of major zoonotic parasitic diseases in South America and global disability-adjusted life years (DALY) estimates (Torgerson and Macpherson, 2011; Global Burden of Disease Collaborative Network, GBD, 2019; World Malaria Report (WHO, 2023a). Country-level burden scores derived from WHO soil-transmitted helminth (STH), schistosomiasis and Chagas disease profiles, and PAHO malaria/leishmaniasis surveillance.

Underreporting, limited access to diagnostics, environmental degradation, lack of sanitization, urbanization and political instability continue to undermine further achievements towards control and elimination of various neglected tropical diseases (NTDs) caused by parasites (Hotez et al., 2008; Recht *et al.* 2017; Global Burden of Disease Collaborative Network, GBD, 2021). Soil-transmitted helminths (STH), schistosomiasis and toxoplasmosis persist across multiple South American nations – particularly Brazil, Bolivia and Colombia – where diagnostic capacity is limited and public health infrastructure remains underfunded (Pan American Health Organization, 2003, 2019; World Health Organization, 2012). It was estimated that in 2012 alone, nearly 50 million preschool and school-age children in Latin America and the Caribbean required treatment for STH (Servián et al., 2024). The burden of zoonotic parasitic infections is heaviest among children, rural populations and immunocompromised individuals (Torgerson and Macpherson, 2011; Lv et al., 2024). In Bolivia, for example, Chagas disease affects 6.1% of the population, while Brazil faces an estimated 41.7 million STH cases and 1.5 million schistosomiasis cases, mostly concentrated in Minas Gerais and Bahia regions (Centre for Disease Control and Prevention, CDC, 2022; Pinazo et al., 2022; Silva et al., 2022). Other common parasitic zoonosis in the Global South include leishmaniasis (∼3.3 million disability-adjusted life years (DALYs)), toxoplasmosis (∼1 million DALYs) and cysticercosis (∼2.8 million DALYs) (Figure 1), where prevalences have been strongly associated with poverty, poor housing and co-endemicity in vulnerable areas (Hotez et al., 2008; Global Burden of Disease Collaborative Network, GBD, 2021; Dubey, 2021; Ta and Blond, 2022; Dantas-Torres, 2024 ).

Tackling this burden requires an integrated ‘One Health’ strategy that bridges human, animal and environmental health. Priorities include (1) Strengthening regional disease surveillance; (2) Expanding access to affordable and accurate diagnostics; (3) Supporting locally driven R&D efforts; and (4) Promoting health education at the community level. Moreover, long-term international funding and equitable research partnerships are essential to sustainably reduce the impact of these neglected diseases (UNESCO, 2015). Using such approaches, national control programmes and international collaboration have led to the elimination of onchocerciasis in the Americas and a 70% reduction in malaria and Chagas disease incidence in Latin American countries (Moncayo and Silveira, 2009; World Health Organization, 2024). In addition, Brazil, Colombia, Peru and Bolivia have significantly reduced malaria incidence in the last 20 years (Recht *et al.* 2017). Brazil, for instance, has achieved an incredible 76.8% decrease in malaria cases between 2000 and 2014, which was accompanied by a reduced DALY rate from 42.5 in 1990 to 3.4 per 100,000 in 2017 (World Health Organization, 2023a). These are examples where investment in surveillance and health polices make control and elimination of NTDs attainable.

## Building the future of parasitology: the role and challenges of early-career researchers in South America

### The central role of early-career researchers

Early-career researchers (ECRs) – someone who is in the initial stages of their research career, i.e., 7–10 years of their last and highest degree, i.e., doctorate (Wellcome) – have an indispensable role in research and development of a country. They design and lead experiments, fieldwork and writing of articles, and often bring bold, interdisciplinary approaches to research fields such as parasitology (Krauss et al., 2023). In South America, their work in developing new strategies for zoonotic diseases diagnostics, control, treatment and vaccine development is increasingly recognized as essential for both regional and global health. Additionally, their collaborations with international research teams provide valuable local insights into global parasitology research, an essential factor in studying parasites that cross borders and impact multiple regions. However, ECRs in South America face a distinct set of structural barriers that impede their permanence in science and impact. These challenges are summarized in Table 1, which outlines the most pressing systemic hurdles for ECRs working in the Global South.

**Table 1.** Systemic challenges faced by South American ECRs

Underfunding and precarity	High research costs	Brain drain and attrition
South American ECRs often operate under fellowship schemes lacking job security and benefits. In Brazil, Postdocs typically receive modest stipends (∼USD 1000/month) without healthcare, leave or pension coverage, compared to >USD 55 000/year with benefits in the USA or EU.	Limited domestic supply chains, high import taxes and currency volatility inflate the cost of reagents and equipment, cost of open access publications, which further restricts research productivity.	More than 50% of PhD graduates exit research within 5 years; others migrate to higher-income regions, weakening institutional continuity and national research capacity.

*Sources*: Docquier and Rapoport, 2012; SciDev.Net, 2021; OECD, 2017; Ciocca and Delgado, 2017; European Commission, 2021; Kwon, 2022; CAPES, 2023; FAPESP, 2024; National Institutes of Health, NIH, 2024.

This *South American ECRs Special Issue in Zoonotic Diseases caused by Helminths and Protozoa* was developed in response to the impact that zoonotic parasitic diseases have in the Global South and in the unique roles that local ECRs play to tackle them, especially when taking into account cultural and language barriers that are faced when working in such localities. Aware of the unique challenges researchers in this part of the world face in relation to funding, we aimed to support these ECRs by offering a platform to highlight their significant work. An Article Processing Charge (APC) charges waiver process was put in place by the publisher, Cambridge University Press & Assessment, to facilitate their publications in *Parasitology*, which is a gold open access journal. This Special Issue reunites 11 research papers that focus on some of the most important zoonotic parasitic diseases in the world, including malaria, leishmaniasis, toxoplasmosis and neurocysticercosis, and span from basic science including field epidemiology and diagnostics, to structural biology and vaccine development, showcasing not only scientific diversity but also the leadership emerging from within the region (Table 2).

**Table 2.** List of the manuscripts included in this special issue

#	Title	Author	Lead country	Main contribution
1	The dsRNA dependent kinase (PKR) inhibits the growth of Leishmania major via NF-kB-mediated genes	Vivarini *et al.*	Brazil	Host–pathogen signalling
2	Plasmodium simium: birth and evolution of a zoonotic malaria parasite species	de Albuquerque *et al.*	Brazil	Evolution and host-switching
3	Preclinical immunogenicity and protective efficacy from a recombinant chimeric protein against visceral leishmaniasis	Lage *et al.*	Brazil	Vaccine development
4	Genetic Diversity of Dioctophyme renale in Southern South America	Arce *et al.*	Argentina	Zoonotic helminth population genetics
5	HMG-CoA reductase as a drug target for the treatment of cestodiases: structural modeling and molecular docking studies	Monteiro Guedes *et al.*	Brazil	Drug discovery and docking
6	Genome-wide association study analysis of single nucleotide variants in L. infantum associated with IL-6 inflammatory response in visceral leishmaniasis	da Silva *et al.*	Brazil	Pathogen genomics
7	How patient, infection, and cysticercus characteristics impact the evolution of Taenia solium larva in the human brain: a unique cyst-level analysis	Shamsunder *et al.*	Ecuador	Clinical parasitology
8	Impact of albendazole treatment on the symptom profile of neurocysticercosis patients 14−16 years following diagnosis	Vilela *et al.*	Ecuador	Real-world therapeutic impact
9	Detection of blastocystis cysts in association with biofilms in a polluted watercourse	López-Arias *et al.*	Argentina	Environmental risk mapping
10	*Toxoplasma gondii* seroprevalence, seroconversion rates, and genetic variability in humans from Uruguay	Valentin-Decuadro *et al.*	Uruguay	Epidemiology and genetics
11	Helminths of zoonotic importance in Tayassuidae and Suidae in Brazilian Midwest: risks for human and domestic animal health	de Souza Ramos *et al.*	Brazil	Zoonosis and Epidemiology

### Funding inequities in NTD research

Globally, funding for NTDs research is limited. As part of the roadmap for 2021–2030, WHO estimates a need for USD 1.6 billion annually to meet elimination goals by 2030 (World Health Organization, 2020) and in 2023, during the Reaching the Last Mile Forum at the Conference of the Parties to the Convention (COP28) in Dubai, new endorsements to the Kigali Declaration mobilized USD 777 million towards NTDs elimination goals. Nevertheless, South America, home to more than 50 million people affected by NTDs, receives a disproportionately small share of global health R&D funding. In 2020, low- and middle-income countries (LMICs) received just 0.6% of noncommunicable disease grants, with similarly sparse NTD support (World Health Organization, 2023b). Brazil alone has invested over USD 230 million in NTD research from 2004 to 2020, but political-economic instability has affected significantly funding towards NTDs and overall research in the country (Melo et al., 2023). A data report available at G-FINDER data portal reveals a 6% decrease in NTDs global funding in 2020 in relation to 2019, a trend that is evidently continuing up to 2023 (DNDi, 2022). Unfortunately, the future does not look promising as the recent US federal budget proposals include a 40% cut to the National Institutes of Health (NIH) and calls for the elimination of the Fogarty International Centre, which plays a key role in building research capacity in LMICs (ASTMH, 2025). These cuts have already led to the suspension of new NIH grants to international partners, and halted programmes such as the USAID Malaria Vaccine Development initiative, potentially reversing hard-won gains in parasitic disease control and vaccine innovation (Dyer, 2025; Science Business, 2025; The Guardian, 2025). Major academic institutions have reported staffing reductions and project losses, highlighting the cascading effects of such funding decisions on global health equity and research advancement (Washington Post, 2025).

Despite the various constraints, South America contributes ∼4–5% of global scientific output (Table 3), led by Brazil (65% of regional production) (UNESCO, 2015; SCImago, 2023). Compared to Asia (∼33%), North America (∼25%) and Europe (∼35%), the region’s output highlights significant scientific contributions coming from the region. Importantly, it is expected that a great proportion of these publications (funding, work and or writing) are led by ECRs and by addressing the structural challenges faced by them these outputs can be improved. This requires not only recognition but also coordinated, long-term investment. In Table 4, the actionable priorities to build a more sustainable, equitable research ecosystem for parasitology in South America are outlined.

**Table 3.** Global regional research outputs by volume and percentage

Region	Publications/year	% of Global output	Notes
Asia	∼600 000–650 000	∼32−34%	Driven by China, India, Japan, South Korea
North America	∼470 000	∼23−25%	USA alone ∼ 450k/year
South America	∼85,000–9 ,000	∼4−5%	Brazil = ∼65% of output
Africa	∼35 000–40 000	∼2%	South Africa, Egypt, Nigeria lead
Oceania	∼50 000–55 000	∼2.5−3%	Australia dominant

*Sources*: Nature Index, 2025; SCImago, 2023.

**Table 4.** A call for action: building a sustainable research ecosystem in South America

Expand equitable ERCs funding mechanisms	Infrastructure not just fellowships	Prioritize ethical authorship and data policies	Integrate ECRs into public health policy
Journals and funders must increase APC waivers, LMIC fellowships and microgrants for early-stage research.	Investment in core facilities and reagent supply hubs will yield multiplicative returns.	Southern researchers must be recognized as equal intellectual contributors, not sample providers).	Embed them in national NTDs programmes and decision-making to allow for research findings to be translated into solutions.

*Sources*: Nakamura et al., 2023; Else, 2025.

### Equitable collaboration as a catalyst for innovation

**Figure 2.**
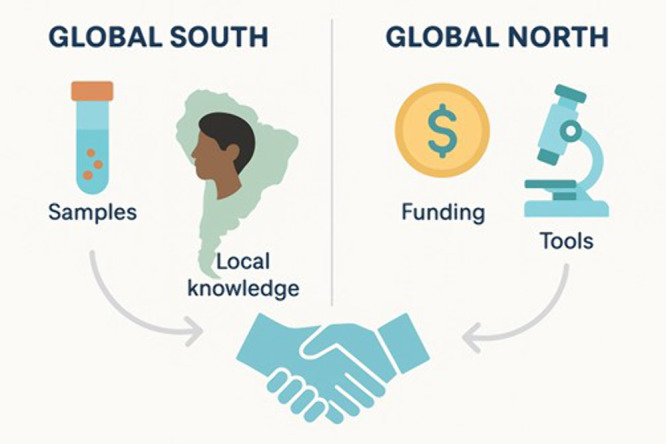
Symbiosis of collaboration. Illustrates mutual contributions of Global North (technology, funds) and South (samples, fieldwork, endemic expertise).

To offset structural limitations, international collaboration is not merely helpful but essential. North–South partnerships provide infrastructure, training and co-authorship opportunities, while South–South collaborations leverage shared disease burdens and cultural proximity (Sakurai, 2015; McManus et al., 2024) (Figure 2). Several international research consortia have demonstrated the transformative potential of equitable collaboration in South America. The NIH Fogarty Global Infectious Disease Program has built sustainable research capacity in Bolivia and Peru by training ECRs and strengthening local surveillance systems (Clark et al., 2020). DNDi-led consortia, working with partners like GlaxoSmithKline and the University of Dundee-UK, have advanced affordable treatments for Chagas disease and leishmaniasis, including paediatric and oral therapies (Drugs for Neglected Diseases Initiative, 2018). Similarly, the CONICET–GCRF network between Argentina and the UK has enhanced helminth drug discovery through joint research and capacity building (DevTracker summary, 2025). These initiatives, which hopefully will continue to exist, underscore how international partnerships grounded in shared leadership and mutual benefit can amplify regional expertise, drive innovation and deliver tangible public health outcomes. To be effective, such partnerships must engage local scientists, communities and policy stakeholders throughout the research life cycle. This ensures relevance, builds capacity and reinforces research sovereignty (Rodrigues, 2021).

## Conclusion: towards an inclusive and equitable future in *Parasitology*

The research presented in this Special Issue exemplifies the resilience, creativity and scientific rigor of the Global South, particularly among ECRs. These studies collectively underscore that global health security hinges on inclusive, equitable and locally anchored research. In an era marked by zoonotic spillover, climate change and antimicrobial resistance, empowering ECRs in endemic regions is not merely a moral imperative but a strategic necessity. As the guest editor, I am proud to highlight the achievements of South American ECRs who, despite limited resources, are advancing parasitological science through innovation, determination and cross-disciplinary approaches. Their work speaks not only to personal commitment but also to a broader regional resilience that merits stronger institutional recognition and structural support. I invite readers to explore the contributions in this issue, connect with the authors and build collaborative networks that reflect the full scope of parasitology’s global diversity. By fostering more equitable research ecosystems, we can help ensure that the future of zoonotic disease research is as dynamic and inclusive as the environments and communities it aims to serve.

## Data Availability

Data supporting the findings of this manuscript are available in the cited bibliography.
